# Phosphorylation of plastoglobular proteins in *Arabidopsis thaliana*

**DOI:** 10.1093/jxb/erw091

**Published:** 2016-03-09

**Authors:** Jens N. Lohscheider, Giulia Friso, Klaas J. van Wijk

**Affiliations:** Section of Plant Biology, School of Integrated Plant Science (SIPS), Cornell University, Ithaca, NY 14853, USA

**Keywords:** ABC1 kinase, chloroplast, FIBRILLIN, meta-analysis, phosphorylation, plastoglobule, proteome.

## Abstract

Plastoglobules (PGs) are plastid lipid–protein particles with a small specialized proteome and metabolome. Among the 30 core PG proteins are six proteins of the ancient ABC1 atypical kinase (ABC1K) family and their locations in an Arabidopsis mRNA-based co-expression network suggested central regulatory roles. To identify candidate ABC1K targets and a possible ABC1K hierarchical phosphorylation network within the chloroplast PG proteome, we searched Arabidopsis phosphoproteomics data from publicly available sources. Evaluation of underlying spectra and/or associated information was challenging for a variety of reasons, but supported pSer sites and a few pThr sites in nine PG proteins, including five FIBRILLINS. PG phosphorylation motifs are discussed in the context of possible responsible kinases. The challenges of collection and evaluation of published Arabidopsis phosphorylation data are discussed, illustrating the importance of deposition of all mass spectrometry data in well-organized repositories such as PRIDE and ProteomeXchange. This study provides a starting point for experimental testing of phosho-sites in PG proteins and also suggests that phosphoproteomics studies specifically designed toward the PG proteome and its ABC1K are needed to understand phosphorylation networks in these specialized particles.

## Highlight

The core plastoglobule proteome consists of 30 proteins and several transiently associated proteins. Based on careful evaluation of published proteomics data, nine of these proteins have one or more serine or threonine phosphorylation sites.

## Introduction

Plastoglobules (PGs) are monolayer lipoprotein particles in plastids that play an important role in plastid biogenesis, senescence and homeostasis, as well as stress responses (reviewed in Eugeni Piller *et al.*, 2012; Brehelin *et al.*, 2007). In chloroplasts, PGs are formed from the outer leaflet of thylakoid membranes, and they remain attached to the thylakoids (Austin *et al.*, 2006). In response to (a)biotic and developmental changes, PGs respond by changes in number and volume; however, underlying mechanisms and control of these dynamic responses are not understood. PGs in chromoplasts are fibrillous (Deruere *et al.*, 1994) and contain mostly carotenoids and enzymes responsible for carotenoid biosynthesis (Ytterberg *et al.*, 2006). PGs in chloroplasts contain tocopherol, various quinones (plastochromonal 8, phylloquinone, plastoquinone), carotenoids and phytylesters (Gaude *et al.*, 2007; Zbierzak *et al.*, 2009; Eugeni Piller *et al.*, 2011; Lippold *et al.*, 2012). In addition to these metabolites, chloroplast PGs contain a specialized core proteome of ~30 proteins (Vidi *et al.*, 2006; Ytterberg *et al.*, 2006; Lundquist *et al.*, 2012a) and several additional proteins that are recruited to PGs under stress conditions (Lundquist *et al.*, 2013). The most abundant PG proteins are several members of the plastid-specific FIBRILLIN family (FBN) (Singh and McNellis, 2011) and members of the ACTIVITY OF *BC*_*1*_ COMPLEX KINASE (ABC1K) family (Lundquist *et al.*, 2012b). FBNs share some sequence homology with lipocalins, which are small proteins involved in binding and transport of small hydrophobic compounds (reviewed in Singh and McNellis, 2011). Arabidopsis has 14 different FBNs of which seven are considered PG core proteins. Loss of function mutants for PG-localized FBN1, FBN2, and FBN4 were affected in (a)biotic stress responses but their specific functions are unknown (Yang *et al.*, 2006; Singh *et al.*, 2010; Youssef *et al.*, 2010). The other FBNs are primarily located in thylakoid membranes (FBN3A,B, 6, 9 and FBN-like) or in the chloroplast stroma (FBN5) (Lundquist *et al.*, 2012a). FBN5 is involved in plastoquinone-9 biosynthesis by interacting with SOLANESYL DIPHOSPHATE SYNTHASES 1 and 2 (Kim *et al.*, 2015). Other PG core proteins include a well-studied TOCOPHEROL (vitamin E) CYCLASE (VTE1) (Porfirova *et al.*, 2002), a key enzyme in tocopherol and plastochromanol biosynthesis, and PHYTYL ESTERASE 1 and 2 (PES1, 2) involved in the formation of phytylesters following cleavage of the phytol tail of chlorophyll and in lipid metabolism (Lippold *et al.*, 2012). An NADP(H) DEHYDROGENASE C1 (NDC1) is likely involved in reduction of oxidized plastochromanols within PGs (Eugeni Piller *et al.*, 2011) and has an essential role as a reductase in phylloquinone biosynthesis (Fatihi *et al.*, 2015). Other PG core proteins have various predicted functional domains, such as methyl-transferases, but their functions have not yet been studied. A comprehensive model for PG functions was proposed based on mRNA co-expression analysis of PG proteins and suggested an involvement of PGs in senescence, prenyl lipid metabolism, plastid biogenesis, redox regulation and the Calvin cycle (Lundquist *et al.*, 2012a). PG-localized ABC1Ks appear to contribute to the coordination of thylakoid membrane and stromal metabolic activities.

ABC1Ks are found in archaea, bacteria and eukaryotes and can be divided into four clades (Lundquist *et al.*, 2012b). The founding member of the ABC1K family is yeast ScCOQ8, which is required for ubiquinone (UQ) synthesis in mitochondria; loss of ScCOQ8 homologues in *E. coli* also causes UQ deficiency (Do *et al.*, 2001). Arabidopsis has 17 ABC1Ks of which eight are likely localized in mitochondria and nine in plastids (Lundquist *et al.*, 2012b). Arabidopsis mitochondrial ABC1K13 can complement the yeast ScCOQ8 mutant (Cardazzo *et al.*, 1998). The Arabidopsis PG core contains six ABC1Ks (1, 3, 5, 6, 7), whereas ABC1K8 (previously named OSA1) is likely associated to inner envelope membranes (Jasinski *et al.*, 2008). ABC1K9, ABC1K1 and ABC1K3 are the most abundant ABC1Ks in PGs. Single *abc1k1* and *abc1k3* mutants as well as the *abc1k1*/*abc1k3* double mutant showed a senescence-like degreening when shifted from low to high light (Lundquist *et al.*, 2013). Metabolite profiling of these single and double mutants revealed altered thylakoid and PG prenyl lipid composition (Lundquist *et al.*, 2013; Martinis *et al.*, 2013a; Martinis *et al.*, 2013b). Additionally, chloroplast enzymes in the jasmonate pathway, as well as PHEOPHYTINASE, which is involved in chlorophyll degradation, were recruited to PGs in *abc1k1*/*abc1k3* under such high light conditions (Lundquist *et al.*, 2013). Two other plastid-localized kinases, ABC1K8 and ABC1K7, were shown to be involved in cadmium tolerance, oxidative stress response and/or lipid metabolism, but it is unclear how they influence these processes (Jasinski *et al.*, 2008; Manara *et al.*, 2014). VTE1 is a likely target of ABC1K1 and/or ABC1K3 activity (Lundquist *et al.*, 2013; Martinis *et al.*, 2013a; Martinis *et al.*, 2013b). These mutant analyses showed that plastid ABC1Ks have important functions in chloroplast functions, but the direct phosphorylation targets for the ABC1Ks are unclear.

Phosphorylation is a very common, reversible post-translational modification that can directly or indirectly regulate protein activity, protein–protein interactions, subcellular localization, and protein stability (Lemeer and Heck, 2009). Mass spectrometry is the method of choice to identify the phosphoproteome (p-proteome) members and their (putative) phosphorylation sites (p-sites) (Junger and Aebersold, 2014). The amount of MS-based p-proteomics data in plants and other species has grown exponentially in recent years and collectively provides a rich source for further analysis (Roux and Thibault, 2013; Vaudel *et al.*, 2016). Meta-analysis of published p-proteomics data from Arabidopsis illustrates this potential but also shows the need for extensive filtering for likely false positives (van Wijk *et al.*, 2014) and spectral evaluation (Lu *et al.*, 2015b). A number of studies specifically focused on Arabidopsis chloroplast protein phosphorylation and the role of thylakoid state transition kinases (STN7, STN8) and thylakoid phosphatases (Shapiguzov *et al.*, 2010; Samol *et al.*, 2012), as well as stromal casein kinase IIα (cpCK2 or PTK), chloroplast sensor kinase (CSK) and pyruvate dehydrogenase kinase (PDK) (reviewed in Bayer *et al.*, 2012; Lundquist *et al.*, 2012b; Rochaix *et al.*, 2012; Schonberg and Baginsky, 2012).

The relative abundance of six ABC1Ks localized in PGs suggests that several proteins within the PG core, or transiently associated with PGs, likely undergo phosphorylation. However, very little is known about the PG phosphorylation status or ABC1K targets. In the current study we collected all publicly available information about (potential) phosphorylation of PG-localized proteins, followed by manual evaluation of underlying spectra of the reported p-sites. None of these studies involved purified PGs but rather total cell or leaf extracts; about 50% of the studies were carried out with cell cultures (for a detailed list of experimental details see van Wijk *et al.* 2014). This indicated phosphorylation of serine and threonine residues (pSer and pThr) of multiple PG proteins, in particular within the FBN family. Several of these sites are conserved across vascular plants suggesting functional importance. These findings are further discussed in the context of the kinase network in chloroplasts.

## Materials and methods

### Collection and evaluation of p-proteomics data

The set of 30 Arabidopsis core PG proteins and additional recruited PG proteins involved in chlorophyll degradation (PPH) and jasmonate biosynthesis (LOX2, LOX3, LOX4, AOC1, AOC2 and AOS) was from Lundquist *et al.* (2012a, 2013). This was complemented with additional members of the ABC1K family (three in plastids and eight in mitochondria) and five stromal or thylakoid FBN family members (Lundquist *et al.*, 2012b; Singh and McNellis, 2011). This set thus contained 55 proteins. Reported phospho-peptides (p-peptides) for this set of proteins were obtained from our recent meta-analysis of Arabididopsis p-protein data (van Wijk *et al.*, 2014) and complemented with the most recent publications by searching the PhosphAt 4.0 database (http://phosphat.uni-hohenheim.de/). Associated metadata, spectra and/or mass peak lists and various search engine scores (MASCOT, Sequest, Inspect) were retrieved from the same sources, as well as publications where these data were published. Spectral data were downloaded from PhosphAt 4.0, PRIDE (http://www.ebi.ac.uk/pride/archive/) or provided by the authors (upon request) of the corresponding publications (Supplementary Table S1 at *JXB* online). Unfortunately, in some cases spectral data were unavailable and we had to rely on published protein scores. For previously unpublished p-peptides listed on PhosphAt 4.0, as well as for peptides with low scores and ambiguous identifications listed in the various publications, a more accurate identification was attempted by repeated Mascot search and manual re-evaluation. Accordingly, the positions of enclosed p-sites were verified by analysis of deposited spectral data and ion fragmentation patterns. Putative p-motifs were assigned based on the p-motifs published in van Wijk *et al.* (2014).

### Analysis of evolutionary conservation of p-sites

In order to identify putative evolutionarily conserved p-sites, sequences homologous to these 55 proteins were obtained by protein BLAST-P search (http://blast.ncbi.nlm.nih.gov/Blast.cgi). Only sequences with the lowest E-values, as well as preferably highest query coverage and sequence identity, were considered. Except for the excavate *Euglena gracilis* (Egr), only species with fully sequenced and released genomes were investigated. The species searched include the higher plants thale cress (*Arabidopsis thaliana*, Ath), poplar (*Popuplus trichocarpa*, Pop), barrel clover (*Medicago truncatula*, Mtr), grape vine (*Vitis vinifera*, Vvi), tomato (*Solanum lycopersicum*, Sly) soy (*Glycine max*, Gma), rice (*Oryza ativa*, Osa), maize (*Zea mays*, Zma), sorghum (*Sorghum bicolor*, Sbi) and oil palm (*Elaeis guineensis*, Egu), the lycopodiophyte *Selaginella moellendorffii* (Smo), the moss *Physcomitrella patens* (Ppa) and several green algae (*Chlamydomonas rheinhadtii*, Cre; *Ostreococcus tauri/lucimarinus*, Ota; *Micriomonas pusilla*, Mpu). Additionally, the extremophile single-cell red algae *Cyanoschyzon merolae* (Cme), *Galdieria sulphraria* (Gsu) as well as carrageen moss (*Chondrus crispus*, Ccr), the glaucophyte *Cyanophora paradoxa* (Cpa) and several cyanobacterial strains (Synechocystis PCC6803, Syn; *Trichodesmium erythraeum IMS101*, Ter; *Nostoc* sp. PCC7120/*Anabaena variabilis* ATCC 29413, Nos; *Prochlorococcus marius*, Pma) were analysed. Secondary algae from the green and red lineage of eukaryotic phototrophs (for chlorarachniophytes: *Bigelowiella natans*, Bna; for the heterokonts: *Phaeodactylum tricornutum*, Ptr; *Thalassiosira pseudonana*, Tps; *Aureococcus anophagefferens*, Aan; *Ectocarpus siliculosus*, Esi; for the cryptophytes: *Guillardia theta*, Gth; for the haptophytes: *Emiliania huxleyii*, Ehu) were included in the sequence analysis. Finally, sequences from human (*Homo sapiens*, Hsa), mouse (*Mus musculus*, Mmu), zebra fish (*Danio rerio*, Dre), the common fruit fly (*Drosophila melanogaster*, Dme), baker’s yeast (*Saccharomyces cerevisiae*, Sce), the slime mould *Dictyostelium discoideum* (Ddi) and *Escherichia coli* (Eco) were also searched. Sequences were aligned using the ClustalW algorithm embedded in the Bioedit free sequence analysis software (http://www.mbio.ncsu.edu/bioedit/bioedit.html). For highly conserved protein families such as the family of carotenoid cleavage oxidases (CCD), flavin reductase-related proteins, as well as the ABC1K and FBN protein families, additional matching sequences were collected and subgroups were assigned by manual comparison.

## Results and discussion

### Assembly and evaluation of the PG p-proteome

The set of Arabidopsis core PG proteins (30) and additional proteins recruited to PGs in the *abc1k1/abc1k3* double mutant (involved in chlorophyll degradation (PPH) and jasmonate biosynthesis (LIPOXYGENASE 1, 2, 3 (LOX2, LOX3, LOX4); ALLENE OXIDE CYCLASE 1 AND 2 (AOC1, AOC2) and ALLENE OXIDE SYNTHASE (AOS)) were from Lundquist *et al.* (2012a, 2013). This set of proteins was complemented with all other members of the ABC1K family (three in plastids and eight in mitochondria) and the remaining five FBN family members located in stroma or thylakoid membrane (Singh and McNellis, 2011; Lundquist *et al.*, 2012b). This set thus contained 55 proteins (Supplementary Table S1). Reported p-peptides for this set of proteins were obtained from our recent meta-analysis of Arabididopsis p-protein data (van Wijk *et al.*, 2014) and complemented with the most recent publications by searching the PhosphAt 4.0 database (http://phosphat.uni-hohenheim.de/). We then tried to evaluate each of the reported p-peptides based on available spectral data, downloaded from PhosphAt, from the public proteome depository PRIDE or ProteomeXchange, or directly obtained from corresponding authors (through individual requests). In a number of cases we could not retrieve the spectral data and we relied on reported significance scores if available, as indicated in Supplementary Table S1. All collected information about reported p-peptides is integrated with the location of p-sites with respect to the (most likely) N-terminus of the mature proteins based on Rowland *et al.* (2015) or PPDB (http://ppdb.tc.cornell.edu) (Supplementary Table S1). In total, 16 of the 30 PG core proteins, as well as transient interacting PPH, AOS and LOX2, four non-PG FBN (FBN3a, 5, 9, 11), non-PG plastid ABC1K4 and ABC1K8, as well as mitochondrial ABC1K13 and ABC1K14 have reported p-sites (Supplementary Table S1). This totals more than 70 potential p-sites in a 71:18:11 ratio for pS:pT:pY. Whereas this seems to suggest that PG proteins and other FBNs and ABC1Ks are frequently phosphorylated, careful inspection of the underlying data (e.g. evidence for p-site within the MS/MS spectrum; see ‘Materials and methods’ and details in Supplementary Table S1) indicates that less than 45% (30 p-sites) could be confirmed with certainty or deemed quite likely (see Supplementary Table S1).

Following a time-consuming and challenging evaluation process of the available p-peptide information and associated spectral data, we considered only 20 non-redundant p-peptides as reliable and additionally three p-peptides as tentative (Table 1). These p-peptides matched to five of the PG FBNs, UNKNOWN 1 and FLAVIN REDUCTASE 1 and 2. A triple phosphorylated peptide was reported for PG-localized VTE1; however, this peptide (partially) maps to the chloroplast transit peptide (cTP) (see below). Furthermore, AOS, which is recruited to PGs during light stress in the *abc1k1*/*abc1k3* double mutant (Lundquist *et al.*, 2013), appears also to be phosphorylated. Tentative phosphorylation evidence was observed for PG-localized NAD(P)-ALDO/KETO REDUCTASE and recruited LOX2. None of the phosphorylation data for the ABC1K proteins fulfilled our criteria, leaving the question of a possible hierarchical phosphorylation between the ABC1Ks unanswered. FBN3A and FBN9, located outside of the PGs appeared also phosphorylated (Table 1). Finally, the p-sites that passed our inspection showed a pS:pT ratio of ~8:1 and no pY sites could be confirmed. In the remainder of this paper, we focus on the selected p-sites and p-proteins listed in Table 1.

**Table 1.** PG-localized and related FIBRILLIN proteins that show reported and (mostly) confirmed p-peptides

Protein i.d.^a^	Protein name	Location^b^	Abundance rank in isolated PG^c^	Module in coexpresion network^d^	Reported p-peptides^e^	p-Site residue number (low confidence p-sites)^f^	Protein length (predicted N-term (exp. N-term))^g^	Motif subtype (based on van Wijk *et al.* 2014)
AT4G0402	FBN1A	Core PG	1	Not in network	VFA**pSpSpSpT**V**pS**VADK	S74, S75, S76, T77, S79	318; A56 (S41, A56, T57)	SxpS, pSxS
AT4G0402	FBN1A	Core PG	1	Not in network	A**pT**DIDDEWGQDGVER	T57	318; A56 (S41, A56, T57)	TD
AT4G2224	FBN1B	Core PG	3	Not in network	A**pT**DTGEIGSALLAAEEAIEDVEETERLKR	T61	310; A60 (A60)	TD
AT4G2224	FBN1B	Core PG	3	Not in network	FAGPLG**pT**NSISTNAK	T188	310; A60 (A60)	—
AT2G3549	FBN2	Core PG	4	III (ABC1K9)	**pS**GPELEESGTR	S105	376; S54 (S54)	SG, SxP
AT2G3549	FBN2	Core PG	4	III (ABC1K9)	SGPELEESG**pT**R	T114	376; S54 (S54)	—
AT3G2340	FBN4	Core PG	2	III (ABC1K9)	LLSVV**pS**GLNR	S96	284; S73 (A54, **S57**, S58)	SG
AT3G2340	FBN4	Core PG	2	III (ABC1K9)	GLVA**pS**VDDLER	S105	284; S73 (A54, **S57**, S58)	—
AT3G2340	FBN4	Core PG	2	III (ABC1K9)	LLYSSAF**pS**SR	S148	284; S73 (A54, **S57**, S58)	—
AT3G2340	FBN4	Core PG	2	III (ABC1K9)	**pS**LGGSRPGLPTGR	S151	284; S73 (A54, **S57**, S58)	xSx, RS
AT3G2340	FBN4	Core PG	2	III (ABC1K9)	SLGG**pS**RPGLPTGR	S155	284; S73 (A54, **S57**, S58)	GS
AT2G4213	FBN7B	Core PG	13	IV	IETPSSTVVETIEYD**pS (C-terminus**)	S299	299; A49 (A49, **V51**)	DS
AT1G3222	FLAVIN REDUCATASE RELATED 1	Core PG	14	Connected to UBIE1	SCVKCTYAEAGL**pS**SASW**pS**APIDIVADVK	(S46), S51	296; A58 (T39)	SxP, SxpS
AT1G3222	FLAVIN REDUCATASE RELATED 1	Core PG	14	Connected to UBIE1	SCVKCTYAEAGLS**pS**ASW**pS**APIDIVADVK	(S47), S51	296; A58 (T39)	SxP, SxpS, pSxS
AT2G3446	FLAVIN REDUCATASE RELATED 2	Core PG	21	II (ABC1K1, 3 ,6)	**pS**YKDLFASVK	S269	280; A52 (M35)	SxxD/E
AT1G0669	NAD(P)-ALDO/KETO REDUCTASE	Core PG	20	II (ABC1K1, 3 ,6)	LGG**pS**DLKVTK	(S54)	377; A32 (A32, V33)	GS, xSDx
AT4G1320	UNKOWN 1	Core PG	15	III (ABC1K9)	TIMVDVEESSSS**pS**DED	S182	185; C57	SDx[D/E]
AT4G3277	VTE1 (cTP)	Core PG	10	Not connected	SISRV**pS**A**pS**I**pSpT**PNSETDK (part of cTP)	S47, S49 (S51, T52)	488; V99 (S49)	pSxS, SxpS, TP
AT5G4265	AOS	Recruited to PG		Not in network	ASG**pS**ETPDLTVATR	S36	518; A33 (S34)	SxpS, GS
AT5G4265	AOS	Recruited to PG		Not in network	A**pS**GSETPDLTVATR	S34	518; A33 (S34)	pSxS, SG
AT3G4514	LOX2	Recruited to PG		Not in network	EFYE**pS**PEK	(S787)	896; R56 (C50)	SP
AT3G2607	FBN3A	Thylakoid		Not in network	GATA**pS**PDDQLR	S92	242; V51	SP
AT4G0003	FBN9	Plastid		Not in network	SSITTDDSLSA**pT**WR	(T87)	212; C26	—

Rows marked in grey indicate lower confidence p-sites. For details, see Supplementary Table S1.

^a^ For proteins with more than one model, we found that model 1 was the best model or that there was no difference in protein sequence with other models. Only in the case of AT2G4213 did we find that model 4 was the most relevant and likely correct model.

^b^ Curated location based on all available information.

^c^ Relative abundance of PG proteins based on label free spectral counting from Lundquist *et al*. (2012).

^d^ Assignment to mRNA based co-expression modules as published in Lundquist *et al*. (2012). Co-expression module I contains ABC1K7, module II contains ABC1K1,3,6, module III contains ABC1K9 and module IV has no kinases. ABC1K5 connects to both module II and III.

^e^ Reported p-peptides in databases or publications. All pS or pT sites are shown in bold.

^f^ p-Site residue number.

^g^ Protein length and predicted or experimental N-terminus. For data see PPDB. Experimental N-termini are based on N-terminal acetylated residues or dimethyl-labelled residues from TAILS experiments (see Rowland *et al.*, 2015). In some cases more than one N-terminus is observed in dimethyl-labelling experiments. Bold indicates the most likely/most frequent experimentally observed N-terminus (based on data in Rowland *et al.* (2015)).

### Phosphorylated PG proteins

The PG core proteome contains seven FIBRILLINS (FBN1A,1B,2,4,7a,7B,8) and most of the observed phosphorylation sites are within this family (FBN1A,1B,2,4,7B). Four of these FBN proteins are also the most abundant proteins in the PG core (Lundquist *et al.*, 2012a) as indicated by the abundance rank in Table 1. The function of these PG-localized FBNs is not known but they have been suggested to play a structural role in PG formation, as well as metabolic transport functions through their lipocalin motif; loss of FBN1, FBN2 or FBN4 affects stress response and results in several metabolite phenotypes (reviewed in Singh and McNellis, 2011).

FBN1A and FBN1B are closely related homologues (E^–130^) and have been suggested to interact with each other via a ‘head–tail’ mechanism *in vivo* using BiFC assays in *Nicotiana benthamiana* leaves (Gamez-Arjona *et al.*, 2014a). FBN1A with two p-peptides has a total of four pS (S74, 75, 76, 78) and two pT (T57 and T77) sites (Table 1; Supplementary Fig. S1). Previously, we observed three different N-termini by mass spectrometry; the most N-terminal residue in isolated PG started at S41, whereas both A56 and T57 were observed with physiological N-terminal acetylation or dimethyl labelling from terminal amine isotopic labeling of substrates (TAILS) experiments (Rowland *et al.*, 2015) and PPDB. T57 was also observed in phosphorylated form (Mascot score 64; the same full tryptic peptide as in Table 1) in isolated thylakoids of the *abc1k1*/*abc1k3* double mutant (unpublished data from the van Wijk lab). Thus the p-sites are all located in the N-terminal region, upstream of the lipocalin domain, suggesting a phosphorylation hotspot. FBN1B was observed by two p-peptides with one pT site (T61) at the N-terminal or penultimate residue and one further downstream (T188) (Table 1; Supplementary Fig. S1). The experimental N-terminus for FBN1B started at A60 based on both methyl labelling and N-terminal acetylation in TAILS experiments. T61 was also observed in phosphorylated form (Mascot score 99) in isolated thylakoids (unpublished data of the van Wijk lab). T57 in FBN1A and T61 in FBN1B are corresponding residues and their conserved phosphorylation suggests functional significance. The highly phosphorylated N-terminal sequence (S74SSTVS78) in FBN1A is absent in FBN1B, indicative of functional differentiation between these two homologues (Supplementary Fig. S1).

**Fig. 1.**
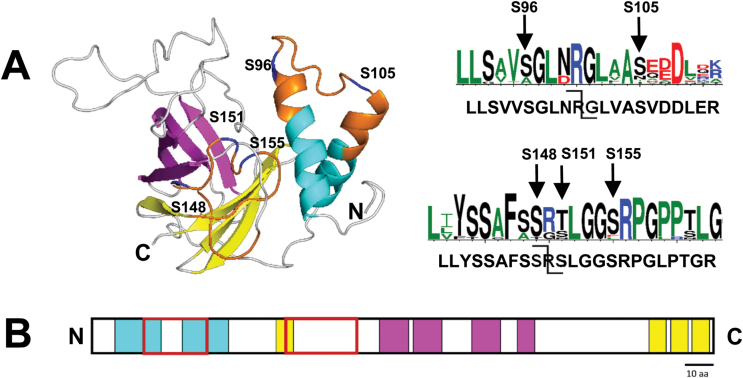
3D model of FBN4 and the location of identified p-peptides. (A) 3D model of FBN4. α-helical structures are shown in cyan, β-sheets in magenta and yellow. Yellow β-sheets represent conserved regions shared among FBN proteins. Identified p-peptides are orange and p-sites are shown in blue. Sequence logos illustrate the degree of evolutionary conservation in 13 land plant species including *Selaginella moellendorffii* and the moss *Physcomitrella patens* with observed p-sites in Arabidopsis indicated by arrows. The model was created using the predicted mature FBN4 protein by I-TASSER (http://zhanglab.ccmb.med.umich.edu/I-TASSER/). (B) Schematic representation of FBN4 domains to illustrate the position of phosphorylated residues. Boxed in red are the sequences covered by the p-peptides. Colour code as in (A).

FBN2 was observed with two full tryptic p-peptides matching to the same sequence, either with pS105 or pT114 located in the N-terminal region upstream of the lipocalin domain. FBN4 has five p-peptides with a total of five pS sites in the N-terminal portion of the protein. Figure 1 shows a 3D model of FBN4 and the location of the identified p-peptides and p-sites. FBN4 has two short α-helical domains connected by a short loop containing pS96 and pS105. The other three p-sites are located in the middle of the proteins in a long flexible loop between β-sheets. The location of the p-sites in these loops rather than within the helical domains or β-sheets is logical since these p-sites are well exposed to the surface to allow access by kinases, thus strengthening the physiological significance of these observations. The two sequence logos (Fig. 1A) illustrate the degree of evolutionary conservation around the p-sites in 13 land plant species including *Selaginella moellendorffii* and *Physcomitrella patens*.

FBN7B (AT3G23400) is represented by five different gene models which differ in N- and C-termini as well as introns. Model 4 has the highest overall peptide count across many experiments in PPDB and Lundquist *et al.* (2012a, 2013) and has an extended C-terminus, similar to models 3 and 5. Models 1, 2 and 4 have a predicted cTP, which is completely absent in models 3 and 5. We consider AT3G23400.4 the most relevant splice form based on all available evidence. Similar to what has been found for many other chloroplast proteins, we observed multiple N-termini (A54, S57 and S58 in models 1, 2 and 4) as evidenced by N-terminal acetylation. The proteoform starting with V51 was by far the most abundant based on N-terminal acetylated and dimethylated (TAILS) peptides, but also N-termini starting with A59 were observed in N-acetylated form. FBN7B is a far less abundant PG protein (~10% compared to FBN1A) and has one p-site (S299) located at the very C-terminus (models 3, 4 and 5). The region is conserved in vascular plants but not in the lycophyte *S. moellendorffii*. Although some of the FBN7 homologues in representatives of the red lineage contain a C-terminal S residue, the general environment at this position seems to be different from the green lineage sequences.

Among the PG core proteins are two FLAVIN REDUCTASE-RELATED proteins (FVR1, 2) with low sequence homology (25% identity). In FVR1, three closely spaced pS sites (S46, S47 and S51) were observed in two p-peptides, with the highest confidence in S46 (see Supplementary Table S1); these pS sites are located only a few residues downstream of the experimentally determined N-terminus (T39) and upstream of the annotated functional domains (see PPDB). There is, however, a discrepancy between the reported p-peptide (SCVKCTYAEAGLSSASWSAPIDIVADVK; Table 1) and the observed N-terminally acetylated N-terminal peptides in our isolated PG particles (Ac-TYAEAGLSSASWSAPIDIVADVK) (128 observations across many experiments). Also, because of the missed cleavage (K37) in the reported p-peptides, we suggest that perhaps these p-peptides are false positives despite the reasonable Sequest scores (11.2 and 10.2; see Supplementary Table S1) or alternatively they represent the unprocessed (phosphorylated) protein still in transit from the cytosol. FVR2 has one reported p-peptide with pS269 in the C-terminal region (the C-terminus is Q280). This C-terminal region is widely conserved in FVR2 homologues in land plants and cyanobacteria, suggesting functional importance of this region.

A multiple phosphorylated peptide (with a missed cleavage for R45) was reported for PG-localized VTE1 (Table 1). The predicted cTP of VTE1 is 98 residues long, thus mapping these p-sites (S47, S49 and low confidence S51 and T52) to the middle of the chloroplast transit peptide (cTP). This location would render these p-sites irrelevant to the function of the mature VTE1 protein within the chloroplast (Table 1). However, our proteomics data for isolated PGs suggest that mature VTE1 starts with S49 (PPDB and Lundquist *et al.* (2012a, 2013)), which would indicate that the reported multi-phosphorylated p-peptide includes a region just upstream and downstream of the observed N-terminus. We also note that peptides matching to cTPs are rarely observed by mass spectrometry even after enrichment for N-terminal peptides; furthermore these cTPs are found in total leaf extracts, rather than in isolated chloroplasts, reflecting highly efficient cTP cleavage and subsequent degradation (Rowland *et al.*, 2015). Therefore, the reported p-peptide cannot be easily explained within these different scenarios. Using radiolabelled ATP, VTE1 was reported to be phosphorylated *in vitro* during incubation with recombinant ABC1K1 or ABC1K3, but the p-site was not determined (Martinis *et al.*, 2013a; Martinis *et al.*, 2013b). Given that tocopherol levels are lower in *abc1k1* and *abc1k1*/*abc1k3* mutants (Lundquist *et al.*, 2013; Martinis *et al.*, 2013a; Martinis *et al.*, 2013b), *in vivo* phosphorylation is a logical scenario, but so far lacks convincing *in vivo* support.

### p-Site motifs and candidate plastid kinases

Protein kinases phosphorylate their peptide substrates by recognizing linear motifs that typically consist of a few key residues surrounding the target amino acid. In our recent meta-analysis and application of *motif-x* and MMFPH prediction algorithms, we identified several motif families for pS and pT sites, which could be divided into more specific types (van Wijk *et al.*, 2014). A subset of these motifs were enriched in chloroplasts/plastids, such as pSP, RxxpS, pSx[D/E], pSxx[D/E] and pSDx[D/E]. Several of these were also observed by other chloroplast/plastid p-proteomics studies in Arabidopsis (Reiland *et al.*, 2009; Schonberg and Baginsky, 2012), as well as other plant species such as orange (Zeng *et al.*, 2014) and rice (Lu *et al.*, 2015a). Among the known plastid kinases, cpCKII has the largest number of (candidate) substrates, and motifs are characterized by one or more acidic residues D or E downstream of pS or pT (Schonberg and Baginsky, 2012; Lu *et al.*, 2015a). However, it should be noted that similar motifs are also observed for other non-chloroplast kinases, such a SLK or CDPK (van Wijk *et al.*, 2014). We evaluated possible p-site motifs in the PG proteome in order to identify potential substrate–kinase relationships. The first conclusion is that there does not appear to be a general consensus motif across the phosphorylated PG core proteins. A few core proteins have downstream acidic D/E residues such as FBN4 (pSxDD), UNKNOWN 1 (pSDxD) and FBN1A,B (pTD). Thylakoid FBN3A and LOX2 have a common pSP motif. Others (FBN1A, FBN1B, FBN4, FVR2, UNKNOWN 1) have one or more positively charged residues (K/R) upstream of the pS or pT site. Based on the currently limited information about plastid kinase specificity and p-site information for PG proteins, kinase–substrate relationships for these phosphorylated PG proteins are not obvious, and will require specific *in vitro* and *in vivo* experiments, including the peptide array experiments as described recently for chloroplast proteins (Schonberg *et al.*, 2014; Schonberg and Baginsky, 2015).

### Distribution of p-proteins across the mRNA-based co-expression modules

mRNA-based co-expression analysis of PG core proteins identified a network with four distinct modules that each contained at least one ABC1K and/or FBN (Lundquist *et al.*, 2012a). It is conceivable that ABC1Ks phosphorylate PG proteins (maybe in cooperation with other kinases such as cpCKII), because they are highly enriched in PGs and at the same time in close vicinity to their putative substrates. It can also be hypothesized that ABC1Ks in a module phosphorylate other members of that module. Module 1 is enriched for proteins involved in senescence, and in addition to ABC1K7, contains three other PG core proteins, but none of these proteins were observed to be phosphorylated. Module 2 is enriched for enzymes of carotenoid biosynthesis as well as plastid proteases and contains three ABC1Ks (1, 3, 6). FLAVIN REDUCTASE 2 in this module has a pS site with two upstream basic residues (R, K) but also an acidic residue downstream (PKRpSYKD). NAD(P)-ALDO/KETO REDUCTASE has a potential p-peptide with a pS in a glycine-rich motif (LGGpSDLK). Given their co-expression and physical proximity, ABC1K1, 3 and 6 are candidates for phosphorylation of these PG proteins but direct or even indirect evidence is lacking. Module 3 has five PG proteins, with FBN2, FBN4, and UNKNOWN 1 likely phosphorylated. In particular FBN4 is highly phosphorylated with four pS sites and one pT site, but the p-sites do not have a shared linear motif (Fig. 1; Table 1). Also FBN2 appears phosphorylated with two fairly close spaced pS and pT sites, whereas the p-site in UNKNOWN 1 is located in a serine rich region with several acidic residues downstream of the p-site (SSSpSDED). ABC1K9 is the only kinase in this module, making it a candidate kinase for phosphorylation of these three PG core proteins. Module 4 has two PG core proteins (UNKNOWN 2 and FBN7B). FBN7B was phosphorylated at the C-terminal serine residue (IEYDpS). This module is enriched for Calvin cycle proteins and plastid biogenesis factors, but lacks an ABC1K.

### What is the location of substrates of PG-localized ABC1K?

The PG-localized ABC1K core proteins are (nearly) exclusively located in the PG. It is therefore logical to assume that their phosphorylation targets are PG core proteins and/or proteins transiently interacting with PGs, such as the stromal enzymes of the plastid-localized jasmonate (JA) biosynthetic pathway (i.e. AOS, LOX2). Identification of proteins transiently interacting with PG is challenging and requires quantitative analysis of the PG proteome and finding the correct conditions to observe these interactions. In the case of the JA pathway as well as PPH, the enrichment was very obvious when studying the *abc1k1*/*abc1k3* double mutant (Lundquist *et al.*, 2013). Stromal fructose biphosphate aldolases (FBPA1,2,3) were reported to be present in isolated PGs (Vidi *et al.*, 2006; Ytterberg *et al.*, 2006), but in subsequent quantitative proteome analysis they were not sufficiently enriched to be considered PG core proteins (Lundquist *et al.*, 2012a). Yet, they may transiently interact with PGs and are therefore candidate targets of ABC1K-driven phosphorylation. It was recently reported that chloroplast starch synthase 4 (SS4; AT4G18240) interacts with FBN1A and PGs (Gamez-Arjona *et al.*, 2014b); however, in none of the mass spectrometry analyses of isolated PGs was SS4, or any of the other three SS proteins, identified (Lundquist *et al.*, 2012a; Lundquist *et al.*, 2013; Ytterberg *et al.*, 2006). We mention this because SS2, SS3 and SS4 have been identified as phosphorylated proteins and because the *abc1k1*/*abc1k3* double mutant, as well as the *abc1k1* single mutant (Martinis *et al.*, 2013b) lacks starch accumulation during light stress, even if comparative proteomics found normal accumulation of stromal proteins involved in the Calvin cycle and starch metabolism (Lundquist *et al.*, 2013). The lack of starch accumulation suggested crosstalk between the PG or thylakoid and Calvin cycle metabolism. As we previously pointed out, the lesion in the Arabidopsis Cycle Electron Flow 1 mutant (CEF1) surprisingly mapped to a chloroplast fructose 1,6-biphosphatase (Livingston *et al.*, 2010a; Livingston *et al.*, 2010b) supporting crosstalk between thylakoid and stromal carbon metabolism. Module 3 (with ABC1K9 and phosphorylated FBN2 and FBN4) and module 4 (with phosphorylated FBN7B) in the PG core co-expression network were highly enriched for Calvin cycle enzymes and redox regulation, again suggesting a functional connection between the PG and stromal (carbon) metabolism (Lundquist *et al.*, 2012a). In conclusion, it is possible that other proteins than those considered the PG core proteome are targets of the PG-localized ABC1K; however this would require at least transient interaction of these targets with the PG. Systematic comparative p-proteome analysis of loss-of-function *ABC1K* mutants, along with systematic *in vitro* phosphorylation assays with recombinant ABC1K proteins, may help identify PG-localized ABC1K targets.

### Lack of ABC1K (auto)phosphorylation and the search for direct ABC1 kinase activity

Direct demonstration of kinase activity of members of the ABC1K family (also named ADCK or UbiB in non-plant species) has been elusive. Furthermore, ABC1K proteins do not show any auto-phosphorylation activity. However, *in vivo* analysis in yeast did link kinase domains in the yeast ABC1K COQ8 and human ADCK3 to phosphorylation and function of several proteins required for ubiquinone biosynthesis in mitochondria (Xie *et al.*, 2011; Xie *et al.*, 2012). The observed correlation between these ABC1 homologues and protein phosphorylation is strong and suggests that these ABC1K proteins are indeed kinases, but there is no direct evidence for their protein kinase activity. A recent paper in which structure–function of human ADCK3 was investigated showed that specific and conserved features of ADCK3 inhibited its protein kinase activity (Stefely *et al.*, 2015). A single point mutation in ADCK3 enabled autophosphorylation. It was suggested that autoinhibition of its kinase activity is important for isoprenyl lipid metabolism and that ADCK3 perhaps phosphorylates prenyl lipids, rather than proteins (Stefely *et al.*, 2015).

### Did this study identify all p-sites in the PG proteome?

This meta-analysis is based upon previously published p-proteomics studies using Arabidopsis cell cultures (~50 of the studies), or tissues from seedlings, leaf rosettes, roots and pollen (see Table 1 in van Wijk *et al.*, 2014), supplemented with additional studies published since February 2013, as uploaded in PhosPhat DB (http://phosphat.uni-hohenheim.de/). None of these studies involved isolated chloroplasts or isolated PGs. Additional specific studies on chloroplast fractions, in particular thylakoids (e.g. Samol *et al.* (2012) and reviewed in Pesaresi *et al.* (2011)), did not identify additional phosphorylated PG core proteins. Preliminary in-house phosphorylation studies on isolated thylakoid proteomes did find several of the p-sites listed in Table 1, but found no evidence for phosphorylation of PG-localized ABC1K (unpublished results). PG proteins are of relatively low abundance, except for the most abundant PG members, FBN1A, 1B, 2, 4 (ranked 1–4) (Table 1). Indeed, the observed p-sites in the PG core proteome are favour these most abundant proteins. It is likely that the current set of available p-proteomics data under-reports phosphorylation events of low-abundance proteins. With the ever increasing sensitivity of modern mass spectrometers, such under-reporting is likely to be alleviated and perhaps additional p-sites in PG core proteins will be identified.

### The importance of submission to community proteomics/mass spectrometry databases

Over the last decade or so, many p-proteomics studies have been published for many species. The size and quality of the published p-proteomics datasets has improved steadily due to vast improvement of speed and sensitivity of mass spectrometers, as well as improvements of p-peptide affinity materials and protocols (Junger and Aebersold, 2014). It is widely recognized in the proteomics community that to increase the utility of these published datasets, it is instrumental that the underlying mass spectrometry and associated meta-data are easily accessible (Roux and Thibault, 2013; Vaudel *et al.*, 2016). Retrieval of published datasets will allow re-analysis of published data, as well as integration of multiple published datasets, as was illustrated in our recent meta-analysis of Arabidopsis p-proteomics studies (van Wijk *et al.*, 2014). However, as is evident from the current study and Lu *et al.* (2015b), spectral evaluation (either manual or using automated specific p-site scoring algorithms) is important to ensure high confidence p-site assignment. PRIDE and ProteomeXchange have emerged as the best supported data repository systems and the process of submission of mass spectrometry data into these databases has recently been streamlined and made much less cumbersome (Vizcaino *et al.*, 2014; Vizcaino *et al.*, 2016). Within the published plant proteomics literature, a significant number of studies did submit their underlying mass spectrometry data to these public databases, which greatly facilitated our re-analysis of the p-proteomics data and evaluation of the underlying spectral support for p-sites. For publications that lacked such submission, this process was much harder and involved contacting the respective authors in the hope that they would be able to provide the relevant mass spectrometry files. Submission of underlying mass spectrometry data to PRIDE/ProteomeXchange should accompany publication of studies that incorporate mass spectrometry-driven proteomics.

## Supplementary data

Supplementary data are available at *JXB* online.

Supplementary Table S1. PG-localized proteins and related ABC1K and FIBRILLINS and their reported p-peptides.

Figure S1. Sequence alignment of FBN1A and FBN1B, their p-peptides, p-sites (in red) and observed N-termini (indicated by arrows).

Supplementary Data
