# Supplementary material for: Phosphorylation of plastoglobular proteins in Arabidopsis thaliana
Source: J Exp Bot. 2016 Mar 9;67(13):3975–84. doi: 10.1093/jxb/erw091 (PMC4915526; doi:10.1093/jxb/erw091)
Supplement: Supplementary Data [file supp_67_13_3975__index.html]

Phosphorylation of plastoglobular proteins in Arabidopsis thaliana — Phosphorylation of plastoglobular proteins in Arabidopsis thaliana — Supplementary Data 

# Phosphorylation of plastoglobular proteins in *Arabidopsis thaliana*

## Supplementary Data

Data files

- supplementary\_figure\_S1.pdf - Supplementary Data
- supplementary\_table\_S1.pdf - Supplementary Data
