# Supplementary material for: Phosphorylation of plastoglobular proteins in Arabidopsis thaliana
Source: J Exp Bot. 2016 Mar 9;67(13):3975–84. doi: 10.1093/jxb/erw091 (PMC4915526; doi:10.1093/jxb/erw091)
Supplement: Supplementary Data [file supp_erw091_supplementary_figure_S1.pdf]

```
Query: AT4G04020.1 (size:318) - FBN1A
Target: AT4G22240.1 (size:310) - FBN1B
Query: 1 - 318  Target: 1 - 310  Evalue: 1E-130

FBN1A MATVPLFTQFPCKTLNPSSSNTKHQSKSPILLPINSINRRSEI---GVSVH-RPDFKIRATDIDDEWGQDGVERVFASSS
      MATV L TQF C+T     S N+K  SK P L+P+ SI  R  I   G++V  R  FK+RATD     G+ G         S
FBN1B MATVQLSTQFSCQTRVSISPNSKSISKPPFLVPVTSIIHRPMISTGGIAVSPRRVFKVRATDT----GEIG--------S

      TVSVADKAIESVEETERLKRSLADSLYGTDRGLSVSSDTRAEISELITQLESKNPTPAPNEALFLLNGKWILAYTSFVGL
       +  A++AIE VEETERLKRSL DSLYGTDRGLS SS+TRAEI +LITQLESKNPTPAP EALFLLNGKWILAYTSFV L
      ALLAAEEAIEDVEETERLKRSLVDSLYGTDRGLSASSETRAEIGDLITQLESKNPTPAPTEALFLLNGKWILAYTSFVNL

      FPLLSRRIEPLVKVDEISQTIDSDSFTVQNSVRFAGPFSTTSFSTNAKFEIRSPKRVQIKFEQGVIGTPQLTDSIEIPES
      FPLLSR I PL+KVDEISQTIDSD+FTVQNSVRFAGP  T S STNAKFEIRSPKRVQIKFEQGVIGTPQLTDSIEIPE
      FPLLSRGIVPLIKVDEISQTIDSDNFTVQNSVRFAGPLGTNSISTNAKFEIRSPKRVQIKFEQGVIGTPQLTDSIEIPEY

      VEVLGQKIDLNPIKGLLTSVQDTASSVARTISNQPPLKFSLPSDNTQSWLLTTYLDKDLRISRGDGGSVYVLIKEGSSLLNP
      VEVLGQKIDLNPI+GLLTSVQDTASSVARTIS+QPPLKFSLP+DN QSWLLTTYLDKD+RISRGDGGSV+VLIKEGS LLNP
      VEVLGQKIDLNPIRGLLTSVQDTASSVARTISSQPPLKFSLPADNAQSWLLTTYLDKDIRISRGDGGSVFVLIKEGSPLLNP
```

Figure S1

Figure S1. Sequence alignment of FBN1A and FBN1B, their p-peptides, p-sites and observed N-termini.
