# Supplementary material for: Phosphorylation of plastoglobular proteins in Arabidopsis thaliana
Source: J Exp Bot. 2016 Mar 9;67(13):3975–84. doi: 10.1093/jxb/erw091 (PMC4915526; doi:10.1093/jxb/erw091)
Supplement: Supplementary Data [file supp_erw091_supplementary_table_S1.pdf]

**Table S1.** PG-localized and related ABC1K and fibrillin proteins and their reported p-peptides.

| Gene id | Protein name | location (a) | reported in Table 1 (based on strength of evidence) | relative abundance in PG and/or leaf (b) | module in coexpresion network (c) | reported p-peptides (d) | p-site residue number (e ) | protein length and predicted/experimental N-terminal residue (f) | p-site within cTP (g) | Source data (h) | Search engine used to identify reported p-peptide | Scores reported to identify reported p-peptide | Re-analysis with Mascot or/and manual evaluation of MS/MS spectrum | p-peptide confidence level following curation (i) | motif subtype (based on van Wijk et al 2014) |
|---|---|---|---|---|---|---|---|---|---|---|---|---|---|---|---|
| AT4G31390.1 | ABC1K1 | core PG | | 6 | II (ABC1K1,3,6) | LDLpTDpTIK | T604, T606 | 682; A80 (T54; S58; A62) | | PhosPhat 4.0 | Mascot | 17 | peptide not found | 4; no | TD |
| AT1G79600.1 | ABC1K3 | core PG | | 7 | II (ABC1K1,3,6) | n.d. | | 711; A43 | | | | | | | |
| AT1G71810.1 | ABC1K5 | core PG | | 18 | connected to II & III (ABC1K1,3,6,9) | n.d. | | 692; A40 | | | | | | | |
| AT3G24190.1 | ABC1K6 | core PG | | 24 | II (ABC1K1,3,6) | n.d. | | 793; V20 | | | | | | | |
| AT3G07700.1 | ABC1K7 | core PG | | 27 | I (ABC1K7) | AIGFpSSSLENHFTGEATQCpYGSKSK | S26, Y41 | 695; A73 (S67) | cTP | Wang X et al., 2013 | Sequest | 3.308 | spectra not available; Generally, values ≥ 1.9 for +1 ions, ≥ for +2 ions, and ≥ 3.75 have been classified by others as significant | 2; maybe | pSxS |
| AT3G07700.1 | ABC1K7 | core PG | | 27 | I (ABC1K7) | LGQLSpSpTRpSDLFPR | S212, T213, S215 | 695; A73 (S67) | | PhosPhat 4.0 | Mascot | 18 | peptide not found | 4; no | xSDx |
| AT3G07700.1 | ABC1K7 | core PG | | 27 | I (ABC1K7) | NLKLIAEpYFQK | Y310 | 695; A73 (S67) | | PhosPhat 4.0 | Mascot | 16 | peptide not found | 4; no | - |
| AT5G05200.1 | ABC1K9 | core PG | | 5 | III (ABC1K9) | LFERMGATpYIK | Y140 | 540; A45 (Q46) | | PhosPhat 4.0 | Mascot | 16 | peptide not found | 4; no | - |
| AT4G19170.1 | CCD4 | core PG | | 9 | II (ABC1K1,3,6) | YVpYAAIGDPMPK | Y481 | 595; S35 (S42) | | PhosPhat 4.0 | Mascot | 99 | peptide not found | 4; no | - |
| AT1G54570.1 | DGAT3/PES1 | core PG | | 11 | I (ABC1K7) | n.d. | | 704; A28 | | | | | | | |
| AT3G26840.1 | DGAT4/PES2 | core PG | | 23 | not in network | pYKIMGGVPVSHFNIpYK | Y498, Y512 | 701; A66 (V38) | | Engelsberger & Schulze 2012; PhosPhat 4.0 | Mascot | 18-33 | peptide not found phosphorylated | 4; no | - |
| AT3G26840.1 | DGAT4/PES2 | core PG | | 23 | not in network | MAVpTVLPpSVpSGLSAVASSSNLRR | T4, S8, S10 | 701; V65 (S37) | cTP | PhosPhat 4.0 | Mascot | 17 | peptide not found | 4; no | pSxS, SxpS, SG |
| AT3G26840.2 | DGAT4/PES2 | core PG | | 23 | not in network | MAVTVLPSVSGLSAVApSpSpSNLRR | S17, S18, S19 | 701; V65 (S37) | cTP | PhosPhat 4.0 | Mascot | 20-21 | peptide not found | 4; no | pSxS, SxpS |
| AT3G26840.3 | DGAT4/PES2 | core PG | | 23 | not in network | MAVTVLPSVSGLSAVASpSpSNLRR | S18, S19 | 701; V65 (S37) | cTP | PhosPhat 4.0 | Mascot | 22 | peptide not found | 4; no | SxpS |
| AT5G41120.1 | Esterase 1 | core PG | | 30 | not in network | n.d. | | 684; P67 (V54) | | | | | | | |
| AT4G04020.1 | FBN1a | core PG | x | 1 | not in network | VFApSpSpSpTVpSVADK | S74, S75, S76, T77, S7 | 318; A56 (S41) | | Reiland et al., 2011 | Mascot | 75-80 | | 1; yes | SxpS, pSxS |
| AT4G04020.1 | FBN1a | core PG | x | 1 | not in network | ApTDIDDEWGQDGVER | T57 | 318; A56 (S41) | | Roitinger et al., 2015; Wang et al., 2013; Reiland et al., 2011; Reiland et al., 2009 | Mascot; Sequest; Inspect | 68-102; 0.887; 2.634 | | 1; yes | TD |
| AT4G22240.1 | FBN1b | core PG | x | 3 | not in network | ApTDTGEIGSALLAAEEAIEDVEETERLKR | T61 | 310; A60 (A60) | | Hoehenwarter et al., 2013 | Mascot, Sequest | 182, - | confirmed pT61 | 1; yes | TD |
| AT4G22240.1 | FBN1b | core PG | x | 3 | not in network | FAGPLGpTNSISTNAK | T188 | 310; A60 (A60) | | Roitinger et al., 2015 | Mascot | 45 | confirmed T188 - good spectrum | 1; yes | - |
| AT4G22240.1 | FBN1b | core PG | | 3 | not in network | AEIGDLITQLEpSK | S120 | 310; A60 (A60) | | Mithoe et al., 2012 | Mascot | Mascot search from PRIDE downloaded spectrum. Score 21 - | x | 3; weak evidence | - |
| AT2G35490.1 | FBN2 | core PG | x | 4 | III (ABC1K9) | pSGPELEESGTR | S105 | 376; S54 | | Roitinger et al., 2015; Nakagami et al., 2010; Sugiyama et al., 2008 | Mascot | 21-43 | | 1; yes | SG, SxP |
| AT2G35490.1 | FBN2 | core PG | x | 4 | III (ABC1K9) | SGPELEESGpTR | T114 | 376; S54 | | Roitinger et al., 2015 | Mascot | 50 | peptide not found | 1; yes | - |
| AT3G23400.1 | FBN4 | core PG | | 2 | III (ABC1K9) | pYpSFSPLTTPK | Y30, S31 | 284; S73 (A54) | cTP | Engelsberger & Schulze 2012; PhosPhat 4.0 | Mascot | 15-24 | peptide not found | 4; no | pSxS |
| AT3G23400.1 | FBN4 | core PG | x | 2 | III (ABC1K9) | LLSVVpSGLNR | S96 | 284; S73 (A54) | | Roitinger et al., 2015 | Mascot | 45 | | 1; yes | SG |
| AT3G23400.1 | FBN4 | core PG | x | 2 | III (ABC1K9) | GLVApSVDDLER | S105 | 284; S73 (A54) | | Reiland et al., 2011; Reiland et al., 2009 | Inspect; Mascot | 1.79/2.178; 46/70 | confirmed S105; excellent covereage | 1; yes | - |
| AT3G23400.1 | FBN4 | core PG | x | 2 | III (ABC1K9) | LLYSSAFpSSR | S148 | 284; S73 (A54) | | Roitinger et al., 2015 | Mascot | 44 | confirmed coverage for pS148 | 1; yes | - |
| AT3G23400.1 | FBN4 | core PG | x | 2 | III (ABC1K9) | pSLGGSRPGLPTGR | S151 | 284; S73 (A54) | | Roitinger et al., 2015 | Mascot | 24-37 | confirmed pS151 | 1; yes | |
| AT3G23400.1 | FBN4 | core PG | x | 2 | III (ABC1K9) | SLGGpSRPGLPTGR | S155 | 284; S73 (A54) | | Roitinger et al., 2015 | Mascot | 24-37 | confirmed pS155 | 1; yes | GS |
| AT3G58010.1 | FBN7a | core PG | | 8 | III (ABC1K9) | n.d. | | 308; A54 | | | | | | | |
| AT2G42130.4 | FBN7b | core PG | x | 13 | IV | IETPSSTVVETIEYDpS | S299 | 299; A49 | C-terminus | Xue et al., 2013 | Sequest | 3.98 | spectrum not available; from Suppl data: pS299 has highest probability | 1; yes | DS |
| AT2G46910.1 | FBN8 | core PG | | 17 | connected to ABC1K5, II, IV | n.d. | | 284; V41 | | | | | | | |
| AT1G32220.1 | Flavin reductase related 1 | core PG | x | 14 | connected to UbiE1 | SCVKCTYAEAGLpSSASWpSAPIDIVADVK | S46, S51 | 296; A58 (T39) | | Wang X et al., 2013 | Sequest | 4.937 | spectra not available; reported Ascore highest for S51 (11.23) and 4.31 for S46 | 1; yes high score | SxP, SxpS |
| AT1G32220.1 | Flavin reductase related 1 | core PG | x | 14 | connected to UbiE1 | SCVKCTYAEAGLSpSASWpSAPIDIVADVK | S47, S51 | 296; A58 (T39) | | Wang X et al., 2013 | Sequest | 3.16 | spectra not available; reported Ascore highest for S51 (10.18) and 2.47 for S47 | 1; yes high score | SxP, SxpS, pSxS |
| AT1G32220.1 | Flavin reductase related 1 | core PG | | 14 | connected to UbiE1 | pSCVKCTYAEAGLSSASWpSAPIDIVADVK | S34, S51 | 296; A58 (T39) | cTP | Wang X et al., 2013 | Sequest | 0.60383 | spectra not available | 4; no low score | SxP, SxpS |
| AT1G32220.1 | Flavin reductase related 1 | core PG | | 14 | connected to UbiE2 | pSCVKCTYAEAGLSSASWSAPIDIVADVK | | 296; A58 (T39) | cTP | Wang X et al., 2013 | Sequest | | spectra not available | 4; no low score | |

| | | | | | | | | | | | | | | | |
|---|---|---|---|---|---|---|---|---|---|---|---|---|---|---|---|
| AT2G34460.1 | Flavin reductase related 2 | core PG | x | 21 | II (ABC1K1,3,6) | pSYKDLFASVK | S269 | 280; A52 (M35) | | Roitinger et al., 2015 | Mascot | 34 | support for S269, but fragment ions are low intensity | 1; yes | SxxD/E |
| AT2G34460.1 | Flavin reductase related 2 | core PG | | 21 | II (ABC1K1,3,6) | YKIMGGVPVSHFNIYK | | 280; A52 (M35) | | Engelsberger & Schulze 2011; Ph | Mascot | 18-33 | this peptide is not found in this protein. The experimental m/z matched to a different peptide. | 4; no | |
| AT3G27110.1 | M48 protease | core PG | | 29 | I (ABC1K7) | n.d. | | 344; A48 | | | | | | | |
| AT1G06690.1 | NAD(P)-Aldo/keto reductase | core PG | (x) | 20 | II (ABC1K1,3,6) | LGGpSDLKVTK | S54 | 377; A32 (A32, V33) | | Engelsberger & Schulze 2012 | Mascot | 17 with neutral loss | peptide with neutral loss (score 21) | 2; probably yes, but weak evidence | GS, xSDx |
| AT1G06690.1 | NAD(P)-Aldo/keto reductase | core PG | | 20 | II (ABC1K1,3,6) | AVGVSNYpSEK | S211 | 377; A32 | | Engelsberger & Schulze 2012 | Mascot | 15-24 | No hit for AVGVSNYSEK with P but 5 matches for K.AVENSPFLEK.L with mascot score 49-65 | 4; no | SE |
| AT5G08740.1 | NDC1 | core PG | | 12 | II (ABC1K1,3,6) | n.d. | | 519; A53 (A2) | | | | | | | |
| AT4G39730.1 | PLAT/LH2-1 | core PG | | 25 | connected to II (ABC1K6) | n.d. | | 181; A19 | | | | | | | |
| AT3G10130.1 | Soul heme-binding protein | core PG | | 16 | not in network | n.d. | | 309; A71 (R50) | | | | | | | |
| AT1G78140.1 | UbiE methyltransferase-related 1 | core PG | | 19 | connected to FRR1 | n.d. | | 355; S41 | | | | | | | |
| AT2G41040.1 | UbiE methyltransferase-related 2 | core PG | | 22 | II (ABC1K1,3,6) | pSRFPSAAIpSAVAPK | S47, S55 | 352; A56 (A53) | cTP | PhosPhat 4.0 | Mascot | 16-19 | peptide not found | 4; no | - |
| AT4G13200.1 | unknown 1 | core PG | x | 15 | III (ABC1K9) | TIMVDVEESSSSpSDED | S182 | 185; C57 | | Roitinger et al., 2015 | Mascot | 91 | spectra not available; but high p-RS site score (99) | 1; yes | SDx[D/E] |
| AT4G13200.1 | unknown 1 | core PG | | 15 | III (ABC1K9) | TIMVDVEEpSpSSSSDED | S178, S179 | 185; C57 | | Wang P et al., 2013 | Sequest | - | | 4. no; no spectral data available; no | SxpS, pSxS |
| AT4G13200.1 | unknown 1 | core PG | | 15 | III (ABC1K9) | TIMVDVEEpSSpSSSDED | S178, S180 | 185; C57 | | Wang P et al., 2013 | Sequest | - | | 4. no; no spectral data available; no | SxpS, pSxS |
| AT4G13200.1 | unknown 1 | core PG | | 15 | III (ABC1K9) | TIMVDVEEpSSSpSSDED | S178, S181 | 185; C57 | | Wang P et al., 2013 | Sequest | - | | 4. no; no spectral data available; no | SxpS, pSxS |
| AT4G13200.1 | unknown 1 | core PG | | 15 | III (ABC1K9) | TIMVDVEEpSSSSpSDED | S178, S182 | 185; C57 | | Wang P et al., 2013 | Sequest | - | | 4. no; no spectral data available; no | SxpS, pSxS, SDx[D/E] |
| AT4G13200.1 | unknown 1 | core PG | | 15 | III (ABC1K9) | TIMVDVEESpSpSSSDED | S179, S180 | 185; C57 | | Wang P et al., 2013 | Sequest | - | | 4. no; no spectral data available; no | SxpS, pSxS |
| AT4G13200.1 | unknown 1 | core PG | | 15 | III (ABC1K9) | TIMVDVEESpSSpSSDED | S179, S181 | 185; C57 | | Wang P et al., 2013 | Sequest | - | | 4. no; no spectral data available; no | SxpS, pSxS, SDx[D/E] |
| AT4G13200.1 | unknown 1 | core PG | | 15 | III (ABC1K9) | TIMVDVEESpSSSpSDED | S179, S182 | 185; C57 | | Wang P et al., 2013 | Sequest | - | | 4. no; no spectral data available; no | SxpS, pSxS, SDx[D/E] |
| AT3G43540.1 | unknown 2 (DUF1350) | core PG | | 26 | IV | n.d. | | 373; T33 | | | | | | | |
| AT1G73750.1 | unknown SAG | core PG | | 28 | I (ABC1K7) | n.d. | | 452; R50 (V38) | | | | | | | |
| AT4G32770.1 | VTE1 | core PG | (x) | 10 | not connected | SISRVpSApSIpSpTPNSETDK | S47, S49, S51, T52 | 488; V99 (S49) | (cTP) | Nakagami et al., 2010 | Mascot | 21 | S51 and T52 are ambiguous; but S47 and S49 confirmed in Suppl Information | 1; yes | pSxS, SxpS, TP |
| AT3G25760.1 | AOC1 | recruited to PG | | | not in network | n.d. | | 254; P79 (A66) | | | | | | | |
| AT3G25770.1 | AOC2 | recruited to PG | | | not in network | HpSPKILK | S97 | 254; P79 (A66) | | Wu et al., 2013 | Maxquant | - | peptide not found | 4; no | SP |
| AT5G42650.1 | AOS | recruited to PG | x | | not in network | ASGpSETPDLTVATR | S36 | 518; A33 (S34) | | Reiland et al., 2009 | Inspect | 1,422 | no spectral data available | 1. yes; high score | SxpS, GS |
| AT5G42650.1 | AOS | recruited to PG | x | | not in network | ApSGSETPDLTVATR | S34 | 518; A33 (S34) | | Reiland et al., 2009 | Inspect | 0.945 | no spectral data available | 1; yes high score | pSxS, SG |
| AT5G42650.1 | AOS | recruited to PG | | | not in network | TGpSK*DLPIR | S49 | 518; A33 (S34) | | Roitinger et al., 2015 | Mascot | 25 | no spectral data available | no spectral data available | GS |
| AT3G45140.1 | LOX2 | recruited to PG | (x) | | not in network | EFYEpSPEK* | S787 | 896; R56 (C50) | | Roitinger et al., 2015 | Mascot | 34 | no spectra available | 2; weak score | SP |
| AT3G45140.1 | LOX2 | recruited to PG | | | not in network | ELNNTTLYApSR | S488 | 896; R56 (C50) | | Wu et al., 2013 | Maxquant | - | very ambiguous; score 21 | 3; unlikely | - |
| AT1G17420.1 | LOX3 | recruited to PG | | | not in network | n.d. | | 919; A53 (S49) | | | | | | | |
| AT1G72520.1 | LOX4 | recruited to PG | | | not in network | n.d. | | 926; A59 (S55) | | | | | | | |
| AT5G13800.1 | PPH | recruited to PG | | | I (ABC1K7) | GWIKHLEpSGGFEALPLLEDpTEEDWEESRIGR | S416, T428 | 484; S47 | | Wang P et al., 2013 | Sequest | - | | no spectral data available | SG |
| AT5G24970.1 | ABC1K2 | plastid | | | not in network | n.d. | | 719; V60 | | | | | | | |
| AT2G39190.2 | ABC1K4 | plastid | | | not in network | RAIPPVpSVGLGHFADVVR | S40 | 814; A25 | | PhosPhat 4.0 | Mascot | 19-99 | only spectrum for low scoring spectrum | 4; no | SxG |
| AT5G64940.1 | ABC1K8 | plastid (IE or Nucleoid) | | | not in network | QVEVpSVTPGGR | S160 | 761; S29 | | Engelsberger & Schulze 2012 | Mascot | 16 | peptide not found | 4; no | - |
| AT1G51110.1 | FBN10 | Thylakoid | | | not in network | n.d. | | 409; C56 | | | | | | | |
| AT5G53450.1 | FBN11/ORG1 | plastid | | | not in network | LNpTTRAQR | T130 | 686; S53 | | PhosPhat 4.0 | Mascot | 17 | peptide not found | 4; no | - |
| AT3G26070.1 | FBN3a | Thylakoid | x | | not in network | GATApSPDDQLR | S92 | 242; V51 | | Roitinger et al., 2015 | Mascot | 68 | no spectra but good score reported | 1; yes | SP |
| AT3G26080.1 | FBN3b | Thylakoid | | | not in network | n.d. | | 234; I46 | | | | | | | |

| | | | | | | | | | | | | | | |
|---|---|---|---|---|---|---|---|---|---|---|---|---|---|---|
| AT5G09820.2 | FBN5 | Stroma | | not in network | TSNLFQPPpSMAASR | S10 | 296; C62 | cTP | PhosPhat 4.0 | Mascot | 20 | 18 | 3; weak evidence | - |
| AT5G19940.1 | FBN6 | Thylakoid | | not in network | n.d. | | 239; V51 | | | | | | | |
| AT4G00030.1 | FBN9 | plastid | (x) | not in network | SSITTDDSLSApTWR | T87 | 212; C26 | | Mithoe et al., 2012 | Mascot | 20 | weak spectral coverage (only 2 b-ions) | 2; possible | - |
| AT1G11390.1 | ABC1K10-a | mitochondria | | not in network | n.d. | | 624 | | | | | | | |
| AT1G61640.1 | ABC1K10-b | mitochondria | | not in network | n.d. | | 621 | | | | | | | |
| AT5G24810.1 | ABC1K11 | mitochondria | | not in network | n.d. | | 1009 | | | | | | | |
| AT4G24810.2 | ABC1K12-a | mitochondria | | not in network | n.d. | | 481 | | | | | | | |
| AT5G50330.1 | ABC1K12-b | mitochondria | | not in network | n.d. | | 479 | | | | | | | |
| AT4G01660.1 | ABC1K13 | mitochondria | | not in network | pSFpTRLVNGVSLVAK | S7, T9 | 623; L11 | mTP | Engelsberger & Schulze 2012 | Mascot | 17-23 | peptide not found | 4; no | - |
| AT1G65950.1 | ABC1K14 | mitochondria | | not in network | IATAVEGVVRpSSR | S59 | 551; A71 | | Engelsberger & Schulze 2012 | Mascot | 17-99 | not found or very weak | 3; unlikely | RS |
| AT2G40090.1 | ABC1K15 | mitochondria | | not in network | n.d. | | 538 | | | | | | | |

(a) Curated location based on all available information. P-plastid, M-mitochondria, C-cytosol, PG - plastoglobule; IE - chloroplast inner envelope membrane

(b) Relative abundance of PG proteins based on label free spectral counting from Lundquist et al 2012.

(c) Assignment to mRNA based co-expression modules as published in Lundquist et al 2012. Co-expression module I contains ABC1K7, module II contains ABC1K1,3,6, module III contains ABC1K9, module IV has no kinases. ABC1K5 connects to both module II and III

(d) reported p-peptides in databases or publications.

(e) p-site residue number

(f) protein length and predicted or experimental N-terminus

(g) p-site within cTP

(h) Source of the reported peptide

(i) Confidence levels based on all available data and manual inspection: 1- probably true; 2-some evidence; 3 - weak evidence; 4 - not identified upon search of original data; 5 - no spectral data available
